# Evaluation of immunogenicity of gene-deleted and subunit vaccines constructed against the emerging pseudorabies virus variants

**DOI:** 10.1186/s12985-023-02051-w

**Published:** 2023-05-23

**Authors:** Hong-liang Zhang, Rui-hua Zhang, Gang Liu, Gui-mei Li, Feng-xue Wang, Yong-jun Wen, Hu Shan

**Affiliations:** 1 Ministry of Agriculture Key Laboratory of Clinical Diagnosis and Treatment Technology in Animal Diseases, College of Veterinary Medicine, Inner Mongolia Agricultural University, Hohhot, 010018 P.R. China; 2 Shandong Collaborative Innovation Center for Development of Veterinary Pharmaceuticals, College of Veterinary Medicine, Qingdao Agricultural University, Qingdao, 266109 P.R. China

**Keywords:** Pseudorabies virus, Mutant strain, Gene-deleted vaccine, Subunit vaccine, Immune evaluation

## Abstract

**Background:**

Pseudorabies (PR) (also called Aujeszky’s disease, AD) is a serious infectious disease affecting pigs and other animals worldwide. The emergence of variant strains of pseudorabies virus (PRV) since 2011 has led to PR outbreaks in China and a vaccine that antigenically more closely matches these PRV variants could represent an added value to control these infections.

**Methods:**

The objective of this study was to develop new live attenuated and subunit vaccines against PRV variant strains. Genomic alterations of vaccine strains were based on the highly virulent SD-2017 mutant strain and gene-deleted strains SD-2017ΔgE/gI and SD-2017ΔgE/gI/TK, which constructed using homologous recombination technology. PRV gB-DCpep (Dendritic cells targeting peptide) and PorB (the outer membrane pore proteins of *N. meningitidis*) proteins containing gp67 protein secretion signal peptide were expressed using the baculovirus system for the preparation of subunit vaccines. We used experimental animal rabbits to test immunogenicity to evaluate the effect of the newly constructed PR vaccines.

**Results:**

Compared with the PRV-gB subunit vaccine and SD-2017ΔgE/gI inactivated vaccines, rabbits (n = 10) that were intramuscularly vaccinated with SD-2017ΔgE/gI/TK live attenuated vaccine and PRV-gB + PorB subunit vaccine showed significantly higher anti-PRV-specific antibodies as well as neutralizing antibodies and IFN-γ levels in serum. In addition, the SD-2017ΔgE/gI/TK live attenuated vaccine and PRV-gB + PorB subunit vaccine protected (90–100%) rabbits against homologous infection by the PRV variant strain. No obvious pathological damage was observed in these vaccinated rabbits.

**Conclusions:**

The SD-2017ΔgE/gI/TK live attenuated vaccine provided 100% protection against PRV variant challenge. Interestingly, the subunit vaccines with gB protein linked to DCpep and PorB protein as adjuvant may also be a promising and effective PRV variant vaccine candidate.

## Background

Pseudorabies virus (PRV) or Suid Herpesvirus-1 (SuHV1) is the causative agent of Pseudorabies (PR) or Aujeszky’s disease (AD) that infects pigs (the only natural host), ruminants including cattle and sheep, carnivores including cats as well as rodents with mortality levels reaching 100% [1]. PRV infection causes acute death of piglets, disrupts breeding, and causes disease of the nervous and respiratory systems in fattening pigs [2]. PRV has spread across multiple species, and there are indications for rare human infections by emerging Chinese PRV strains that may possibly result in endophthalmitis and encephalitis [3, 4]. PR control in China had been relatively stable until the emergence of PRV variant strains in 2011 where infections occurred in pigs even though the animals had been immunized with the Bartha-K61 strain [5, 6]. The Bartha-K61 vaccine appeared to provide only suboptimal protection against these variants [7, 8], although other studies do show adequate protection against such variants by the Bartha-K61 vaccine strain [9, 10]. Irrespective, the generation of vaccines that antigenically more closely match emerging variant PRV strains may represent an added value to control these infections.

The PRV glycoprotein gE is a virulence protein of PRV that contributes to neuronal spread [11]. Deletion of the gE gene (encoded by US8) significantly attenuates virulence and the use of this variant as a vaccine strain displayed a good safety profile [12]. In addition, almost all wildtype PRV strains express gE, which is used for differential diagnosis of wildtype PRV infections [13]. The gI and gE complexes act together with gC to mediate virus formation and disruptions in these genes negatively affect virus replication and virulence [14, 15]. The PRV thymidine kinase (TK) is also used to determine the virulence of the PRV virus, as it is involved in viral replication and latent infection, and plays an important role in central nervous system proliferation [12]. Therefore, deletion of gE, gI, and TK genesare often used to construct PRV live attenuated vaccine strains. The PRV gB protein had been proven to induce neutralizing antibodies in animals, and gB protein has been tested as a subunit vaccine target and diagnostic antigen [16].

In previous studies, we isolated and identified a highly virulent PRV variant SD-2017 strain. Sequence analysis of this variant indicated it was closely related to PRV variants that have recently emerged in other areas of China [17]. In the current study, we used the SD-2017 genomic sequence information to construct an inactivated ΔgE/gI vaccine and a live attenuated vaccine lacking gE/gI/TK. We additionally used baculovirus to purify gB and the *Neisseria meningitidis* PorB protein to prepare a novel subunit vaccine PRV gB-DCpep. Rabbits are one of the most sensitive experimental animals for PRV and have specific itchy symptoms after the onset of disease. Therefore, rabbits are often vaccinated to diagnose PRV and evaluate vaccine quality. So we examined the pathogenicity, immunogenicity and clinical protection of these PRV vaccines in rabbits and the primary objective was to develop new live attenuated and subunit vaccines against PRV variants.

## Materials and methods

### Virus and cell lines

The wild-type PRV virulent SD-2017 strain was isolated in 2017 from a pig farm in Shandong, China and was preserved in the Chinese General Microbiological Culture Collection Center (No. 22,047). The SD-2017 gE gene (Acc. No. MW535262) was used to identify the PRV variant strain circulating in China. In order to avoid the mutation of parent strains caused by passing culture, we used SD-2017 strain of the lower generation (F10) in this study. This strain had limited adaptability on PK15 cells and had no significant difference in virus titer and plaque size compared with Bartha-K/61. The PRV Bartha-K61 vaccine strain was obtained from Shandong Huahong Biological Engineering Co., LTD, and was stored in our lab. Bartha-K/61 strain has a deletion of 3,489 bp nucleotides at positions 122,560 − 126,049, including part of the gI gene, all the gE and Us9 genes, and part of the Us2 gene. PK15 porcine kidney cells were cultured in DMEM (Gibco, Grand Island, NY, USA) containing 10% fetal bovine serum (Gibco) at 37 °C in 5% CO_2_ in a humidified incubator. Protein purification utilized Sf9 cells (ATCC CRL-1711) to propagate recombinant baculovirus and were cultured in Grace insect medium (Gibco) containing 2% FBS at 27 °C.

### Primers and plasmids

Homologous recombination technology was used to construct the gE/gI and TK gene deletions of SD-2017. The pBluescript II KS(+) plasmid was used as the backbone and PCR amplicons of gE/gI and TK were cloned into the plasmid using standard techniques (Table 1). The complete EGFP gene was inserted between the two homology arms for screening of recombinant viruses. Recombinant plasmids pBKS-gIL/gER and pBKS-gIL/gER-EGFP, pBKS-TKL/R and pBKS-TKL/R-EGFP were constructed, respectively.

**Table 1:** Primers used for PRV gE/gI gene and TK gene knockouts

Gene	Primer name	Primer sequence (5’→3’)	length (bp)
gI-L	gI-LF	GGGGTACC(KpnI)GACGCCGCACCGCCCCTTC	796
	gI-LR	CCGCTCGAG(XhoI)GGCACCAGGGCCGTCAGCAC	
gE-R	gE-RF	CCCAAGCTT(HindIII)CGCGCCCGTGGAACCCGTA	1162
	gE-RR	GGACTAGT(SpeI)GCCCCGTCCATCAGCGTGACCA	
TK-L	TK-LF	GGGGTACC(KpnI)CCGCCTTATCATCCCCGCTCCCC	694
	TK-LR	CCGCTCGAG(XhoI)CCATCGGCTCGGGCACGTACAG	
TK-R	TK-RF	CCCAAGCTT(HindIII)GGTGTGACCCTCGCCCCTC	854
	TK-RR	GGACTAGT(SpeI)TCGCGCATCGTCTGGTGCAT	
EGFP	EGFP-F	CCGCTCGAG(XhoI)ACTCTGTTCGACACGGACAC	1709
	EGFP-R	CCCAAGCTT(HindIII)ATCTCCGACGTGAAGGCGCA	

### DNA extraction and transfection

A commercially plasmid purification kit (Omega Biotec, Norcross, GA, USA) was used to prepare plasmid DNA. The PRV SD-2017 strain was cultivated in PK15 cells and virions were purified in 20–60% sucrose density gradients by centrifugation at 130,000 × g at 4 °C for 2 h. Viral DNA was purified from infected cells or purified virus using the DNAiso Reagent (Takara, Dalian, China). Transfections were carried out using a calcium phosphate cell transfection kit (Thermo Scientific, Pittsburg, PA, USA) following the manufacturer’s instructions.

### Generation of PRV mutants

**Fig. 1:**
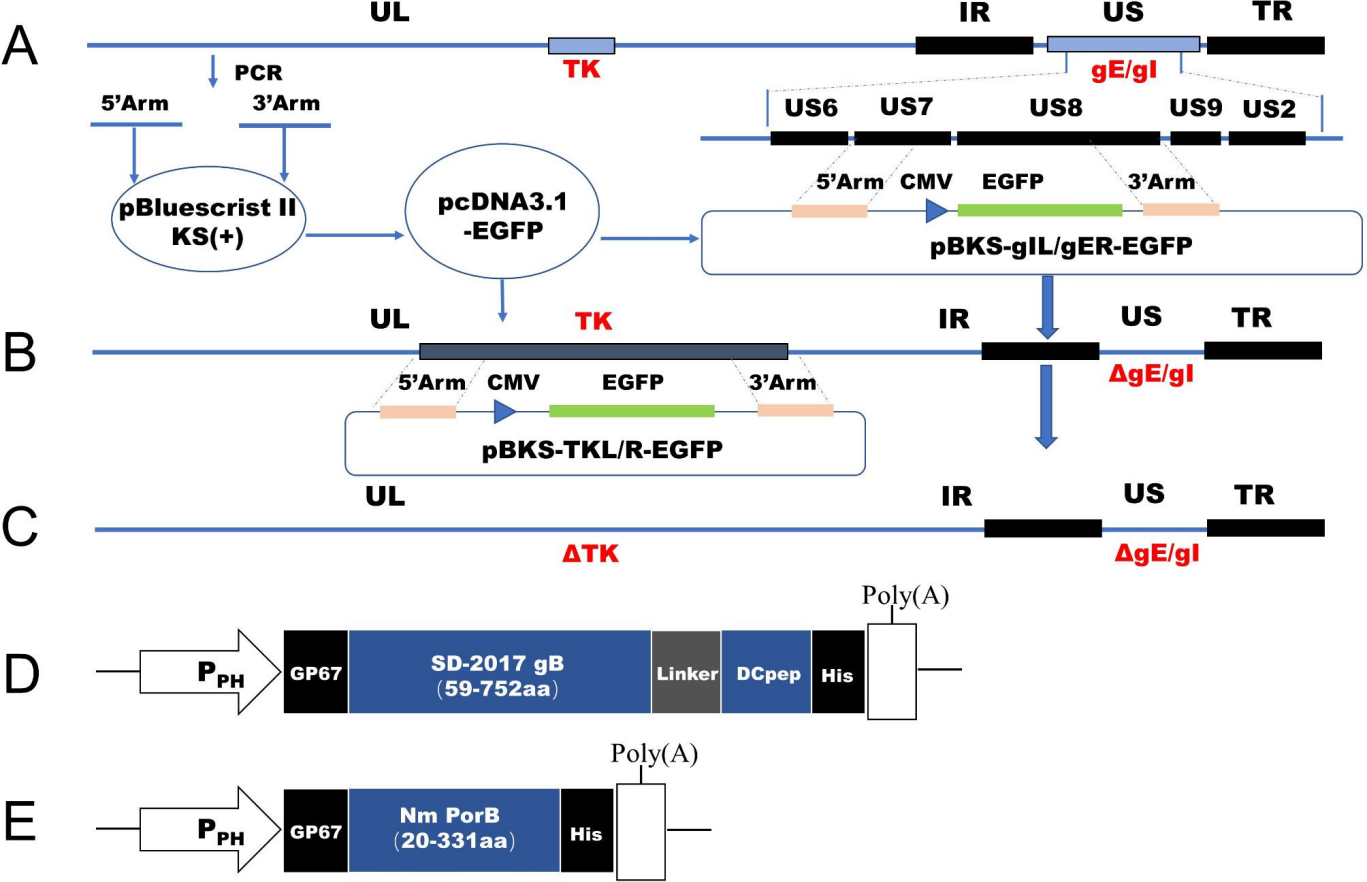
Strategy for the construction of gene-deleted and subunit vaccines strains. Schematic representations of the **(A)** SD-2017 genome and **(B)** the SD-2017ΔgE/gI genome. **(C)** Sites of gene replacements and the genome organization of the SD-2017ΔgE/gI/TK strain. Schematic representation of the **(D)** PRV SD-2017gB-DCpep and **(E)** MC PorB recombinant proteins

A homologous recombination method (Fig. 1A-C) was used to generate viral mutants as previously described [18]. Briefly, SD-2017 gDNA and the plasmid pBKS-gIL/gER-EGFP were co-transfected into PK15 cells (5 × 10^5^ cells/well) and green fluorescent plaques were identified under a fluorescent microscope. These plaques were picked and recombinant virus was obtained through plaque purification. Identification of gE/gI gene deletions and the presence of gB were performed using PCR and sequence analysis (Table 1). Multiple rounds of plaque purification resulted in the identification of a reconstructed virus SD-2017ΔgE/gI-EGFP lacking the gE/gI gene and expressing the eGFP gene (green fluorescence) was obtained. SD-2017ΔgE/gI-EGFP DNA and plasmid pBKS-gIL/gER were then co-transfected into PK15 cells and non-fluorescent viral plaques were purified and identified (see above) to obtain the recombinant virus SD-2017ΔgE/gI lacking eGFP. The same strategy (see above) was used to obtain the recombinant virus SD-2017ΔgE/gI/TK.

### PRV growth kinetics and plaque size determination

Growth kinetics of PRV in PK15 cells was examined as previously described [19]. In brief, PK15 cells were grown to 80% confluence in a 24-well plate and infected with SD-2017 (F10), SD-2017ΔgE/gI (F11), SD-2017ΔTK/gE/gI (F11) and Bartha-K61 at a multiplicity of infection of 1 (MOI = 1.0). Virus titers were expressed as 50% tissue culture infection dose (TCID_50_) and were recorded as TCID_50_/mL [20]. All experiments were repeated at least 3 times and the average titers were calculated and kinetic growth curves for each tested virus were constructed. Plaque sizes were measured from 500 TCID_50_ of PRV inoculated into PK15 cells in a six-well plate for 48 h that was then overlayed with 1% agarose medium and the plaque sizes was measured 1 h later. For each virus, 100 plaques were randomly selected and their sizes was determined from photomicrographs using ImageJ 2.7 software (https://imagej.nih.gov/ij/).

### Expression of PRV gB and ***N. meningitidis*** PorB fusion proteins in recombinant baculovirus

Plasmids expressing the gB-DCpep fusion protein of SD-2017 (gB Acc No. MW535262) and the PorB protein of *N. meningitidis* (Acc. No. AE002098.2) were constructed using standard recombinant techniques. In brief, PCR-based gene assembly methods were used to generate gene fusions that were verified by sequence analysis (Fig. 1D and E). Purified DNA of the fusion constructs gB-DCpep and PorB were digested with restriction endonuclease and cloned into plasmid vector pFastBac 1 to construct a baculovirus transfer vector yielding pFastBac1-gB-DCpep and pFastBac1-PorB, respectively. A commercial kit (Bac-to-Bac, Thermo) was used to produce the recombinant baculovirus strains Ac-gB-DCpep and Ac-PorB using instructions provided by the manufacturer. Sf9 cells in logarithmic growth phase were infected with Ac-gB-DCpep and Ac-PorB at MOI = 1.0 and cultured at 27 °C for 60 h. Proteins were extracted from the culture supernatants as previously described and analyzed using Western blotting [9]. In brief, protein samples were separated using 12% sodium dodecyl sulfate polyacrylamide gel electrophoresis (SDS-PAGE) and then electro-transferred to a polyvinylidene fluoride membrane and incubated in Tris and Tween 20 (TBS-T) containing 1:5000 dilution of anti-His monoclonal antibody (Proteintech, 66005-1-Ig, USA) for 2 h at room temperature. HRP-labeled goat anti-mouse antibody (Jackson, Bar Harbor, ME, USA) was used as the secondary antibody, the membrane was incubated for 1 h at room temperature and washed 3 × in TBS-T, and protein bands were visualized using an ECL chemiluminescence system (Vilber, Fusion FX6, France).

PRV gB-DCpep and PorB proteins were purified from Sf9 cells using an AKTA protein purification system equipped with a nickel affinity chromatography column (GE Healthcare, Marlborough, MA, USA) according to the manufacturer’s instructions. Purified proteins were confirmed using 12% SDS-PAGE quantified using a BCA protein assay kit (Vazyme, E112-01, Nanjing, China).

### Determination of LD_50_

Healthy young SPF rabbits weighing 2.0–2.5 kg were randomly divided into 5 rats in each challenge dose group, and were inoculated with SD-2017 (10^2^ − 10^5^ TCID_50_/mL), SD-2017ΔgE/gI (10^3^ − 10^5^ TCID_50_/mL) or SD-2017ΔgE/gI/TK (10^4^− 10^6^ TCID_50_/mL) to determine LD_50_ and evaluate the virulence of each PRV strain. Rabbit was inoculated intranasally with 0.5 mL of PRV virus and 0.5 mL of DMEM in the control group. The clinical signs and survival rate of each group were observed daily over 14 d following inoculation and the mental state, feeding, itching, body temperature and other clinical signs of each group were recorded. The Karber method was used to calculate the LD_50_ of each strain [21]. All rabbit experiments were performed in Shandong Huahong Biological Engineering Co., LTD (Huahong), and accordance with the protocols approved by the Animal Care and Ethics Committee of the Huahong, under the number 201,907,003. After challenge, rabbits with serious clinical symptoms of depression, pruritus and anorexia were judged as the humanitarian endpoint. Rabbits were euthanized and the death was counted as a statistical result. All surviving rabbits were euthanized at the end of the experiment.

### Immunization and challenge of rabbits

Healthy young SPF rabbits weighing 2.0-2.5 kg were randomly divided into 7 groups (n = 10). The rabbits in each group were immunized by intramuscular injection of the vaccine according to Table 2. DMEM as blank control. Three weeks after vaccination, the same dose was used for booster immunization. Three weeks after the final immunization, the immunized rabbits were infected through the nose with the virulent SD-2017 strain (100 LD_50_). The clinical symptoms and survival rates were recorded for 14 days. At 14 days post-challenge (dpc), all surviving rabbits were euthanized, brain, lung, liver, and kidney samples were collected for histopathological analysis.

**Table 2:** Groups of PRV vaccines in rabbit immunity test

Group	Name	Vaccine antigen	Category	Adjuvant
A	PRV-gB	50 µg gB-DCpep	Inactivated	/
B	PRV-gB + PorB	50 µg gB-DCpep	Inactivated	100 µg PorB
C	SD-2017ΔgE/gI(-) + 206	10^5.0^ TCID_50_ SD-2017ΔgE/gI	Inactivated	1 dose of 206
D	SD-2017ΔgE/gI(-) + PorB	10^5.0^ TCID_50_ SD-2017ΔgE/gI	Inactivated	100 µg PorB
E	SD-2017ΔgE/gI/TK(+)	10^5.0^ TCID_50_ SD-2017ΔgE/gI/TK	Live	/
F	Bartha K61(+)	10^5.5^ TCID_50_ Bartha K61	Live	/
G	DMEM	0.5 mL DMEM	/	/

(+), meant live vaccine; (-), inactivated vaccine; 206, ISA 206 adjuvant

### Evaluation of humoral and cellular immune response post immunization

PRV-specific antibodies (total IgG, IgG1, IgG2a) in the serum were determined using indirect ELISA. In brief, purified gB protein was coated on a 96-well microtiter plate at 0.5 µg per well in the carbonate buffer pH 9.5 at 4 °C overnight and then blocked using 1% bovine serum albumin at 37 °C for 1 h. Serially-diluted serum samples were added and incubated at 37 °C for 1 h followed by the addition of HRP-conjugated goat anti-rabbit IgG (1:5000), IgG1 (1:5000) and IgG2a (1:5000) to measure PRV-specific antibody subtypes. The antibody endpoint titer was determined based on the highest dilution that produced an OD_450_ twice that of the naive group without dilution.

Serum neutralization tests used serum samples diluted in DMEM in 96-well plates to which 50 µL of 200 TCID_50_ SD-2017 virus was added to each well and incubated at 37 °C for 1 h. The same volume of PK15 cell suspension was added to each well, and the PRV-specific neutralizing antibody titers were calculated 3 days later and expressed as the reciprocal of the highest dilution inhibiting PK15 cell infection.

Lymphocyte proliferation was analyzed using an MTS assay kit (Abcam, ab197010, Shanghai, China). Peripheral blood mononuclear cells (PBMC) were prepared using peripheral blood lymphocyte separation solution (Solarbio, P8760, Beijing, China). In brief, 100 µL PBMC in RPMI 1640 / 10% FBS were added to a 96-well plate at 4 × 10^6^ cells/mL. Concanavalin A (10 µg/mL) was added and the incubated at 37 °C in 5% CO_2_ for 72 h. Following this 20 µL of MTS (5 mg/mL) were added to each well and incubated 4 h at 37 °C. The stimulation index (SI) was calculated according to the following formula: SI = (OD value of the immune group-OD value of the control group) / (OD value of the negative control group-OD value of the control group). Th1 (IFN-γ) and Th2 (IL-6) cytokine levels in serum were measured using commercial ELISA kits (Jingmei, Yancheng, China) according to the instructions of the manufacturer.

### Necropsy and pathological examination

Rabbits that reached the humanitarian endpoint and those that survived after the end of the experiment were euthanized by injecting excess pentobarbital sodium. The brain, liver, lung, and kidney organs were taken, fixed in 10% formalin and embedded in paraffin. The buried tissue samples were cut into 4 μm-thick sections, stained with HE and pathological changes were observed using light microscopy.

### Statistical analysis

The data were descriptively analysed using SPSS 23.0 (SPSS Inc, Chicago, IL, USA) for Windows and Excel 2010. The normal distribution was checked with the Shapiro-Wilk test. All data were expressed as the mean ± standard deviation (SD). The Student’s T-test was used to compare group means. One way ANOVA followed by Tukey’s test was used for multiple comparisons. Statistical significance was set at p < 0.05.

## Results

### Characterization and growth characteristics of PRV virulence gene deleted strains

**Fig. 2:**
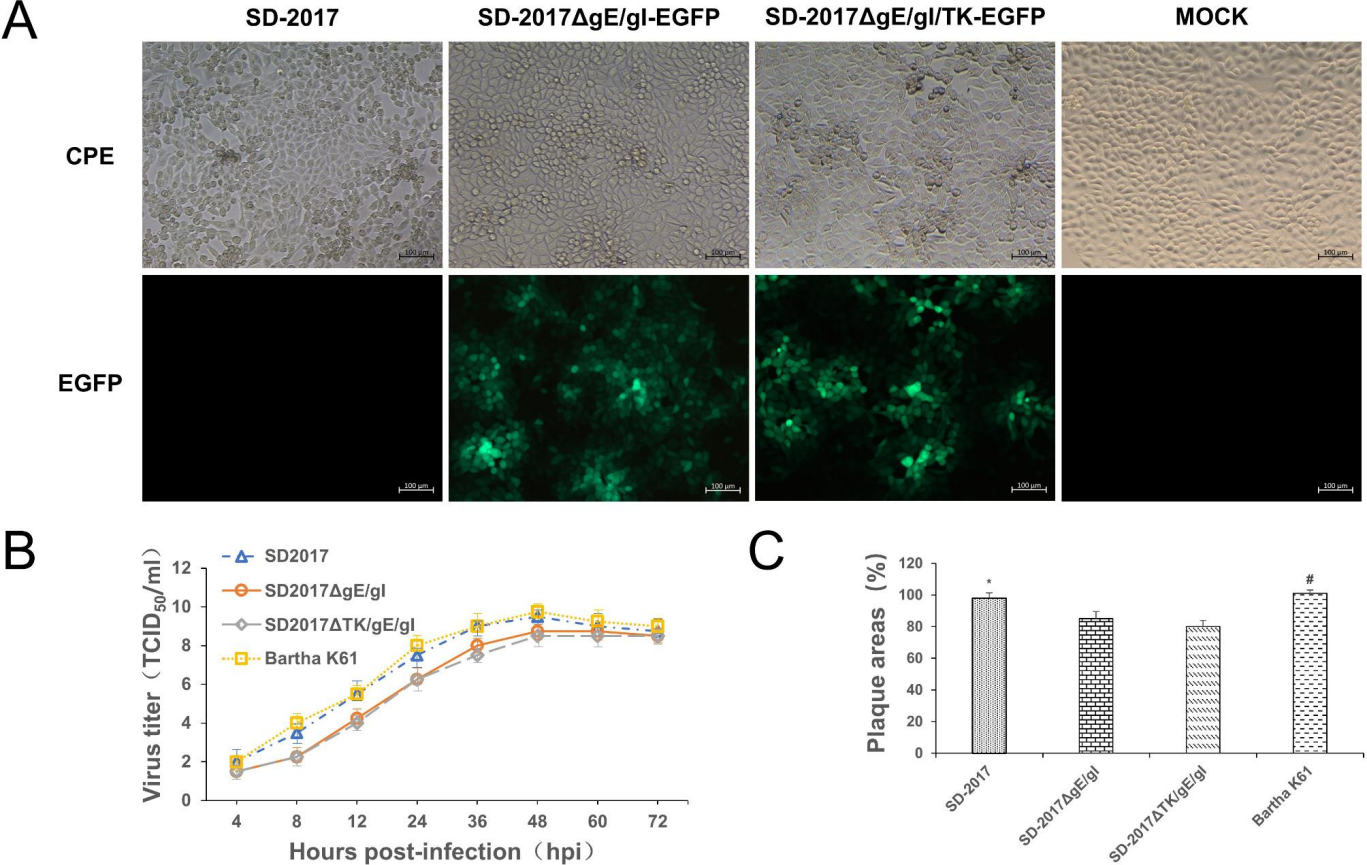
Identification of PRV gene deletion strains. **(A)** Cytopathic effect and eGFP expression of SD-2017ΔgE/gI-EGFP- and SD-2017ΔgE/gI/TK-EGFP-infected cells **(B)** Viral growth curves for SD-2017, SD-2017ΔgE/gI, SD-2017ΔgE/gI/TK and Bartha K61 strains **(C)** Plaque sizes of the indicated viruses

In this study, pBKS-gIL/gER-EGFP and PRV genomic DNA were co-transfected in PK15 cells at a ratio of 3:7 for 48 h. We could identify a large number of green fluorescent plaques in the cells that showed obvious CPE. A total of 6 rounds of virus plaque purification were performed to obtain SD-2017ΔgE/gI-EGFP that possessed the gE/gI knockout and expressed eGFP. The SD-2017ΔgE/gI-EGFP genomic DNA and pBKS-gIL/gER were then co-transfected into PK15 cells, and 10 rounds of screening and purification of viral plaques lacking green fluorescence resulted in the identification and purification of a gE/gI knockout lacking eGFP (SD-2017ΔgE/gI). Using a similar strategy, we screened and purified SD-2017ΔgE/gI/TK-EGFP (Fig. 2A) and SD-2017ΔgE/gI/TK. PCR tests indicated that the gB gene was detected in the genome of SD-2017ΔgE/gI/TK and the gE/gI/TK genes were absent, which consistent with sequence results. Additionally, the growth kinetics of SD-2017ΔgE/gI/TK and SD-2017ΔgE/gI were slightly slower than that of the parental strain SD-2017 and the traditional vaccine strain Bartha K61 (Fig. 2B). The titers of the two recombinant viruses were 10^8^ TCID_50_/mL after culturing for 60 h, but the plaque sizes were smaller than the complete strains, indicating that these genetic manipulations adversely affected PRV proliferation (Fig. 2C).

### Expression and purification of recombinant protein PRV gB-DCpep and PorB

**Fig. 3:**
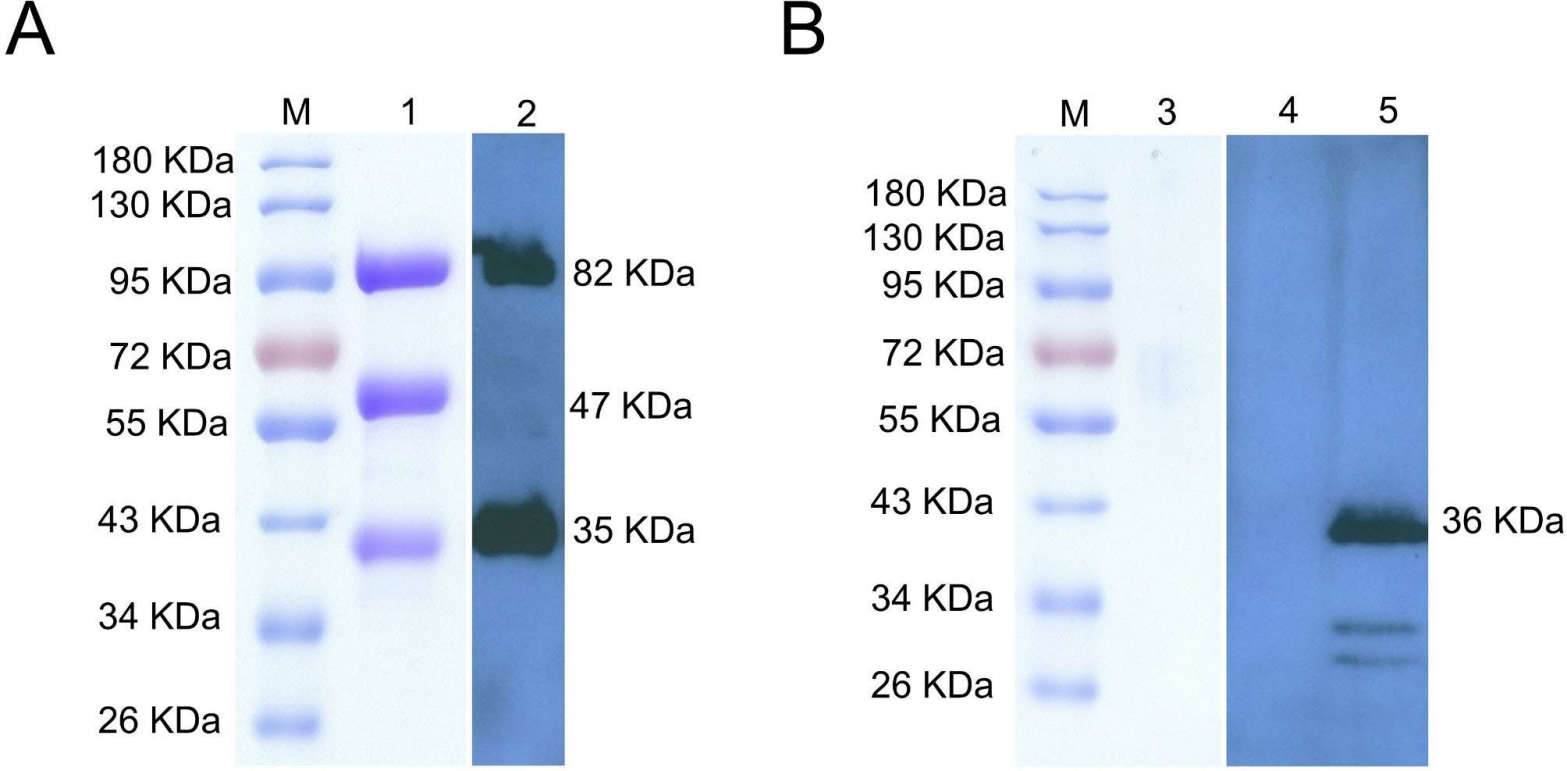
Detection and purification of PRV gB-DCpep and PorB proteins. **(A)** PRV gB-DCpep **(B)** PRV gB-PorB. Lane 1, purified gB-DCpep protein using SDS-PAGE. Lane 2, gB-DCpep protein from culture supernatant of sf9 cells using Western blot and anti-His primary antibody. Lane 3, negative control. Lane 4, culture supernatant of non-infected sf9 cells. Lane 5, PorB protein from sf9 cells detected using anti-His Western blotting

The recombinant baculovirus Ac-gB-DCpep and Ac-PorB genes were sequenced to ensure unintended mutations had not occurred. These constructs were then used to generate PRV gB-DCpep and PorB proteins from P3 generation-infected sf9 cells that were affinity purified using Ni-NTA chromatography. Western blot analysis of the Ac-gB-DCpep generated protein bands at 82, 47 and 35 kDa from stained gels, and the anti-His antibody detected bands at 82 and 35 kDa. The 82 kDa band represented the full-length PRV gB-DCpep protein and the 47 kDa was an N-terminal degradation fragment, while the 35 kDa represented the C-terminal degradation fragment that still reacted with the anti-His antibody (Fig. 3A). In sf9 cells infected with recombinant baculovirus Ac-PorB, a His-reactive band was detected at 36 kDa from the whole cell sample, but was absent from the supernatant. In contrast, no specific protein was produced in negative control cells (Fig. 3B). The results demonstrated that the PRV gB-DCpep and PorB proteins were confirmed and purified.

### Virulence of PRV and immunity of gene deleted PRV strains

**Fig. 4:**
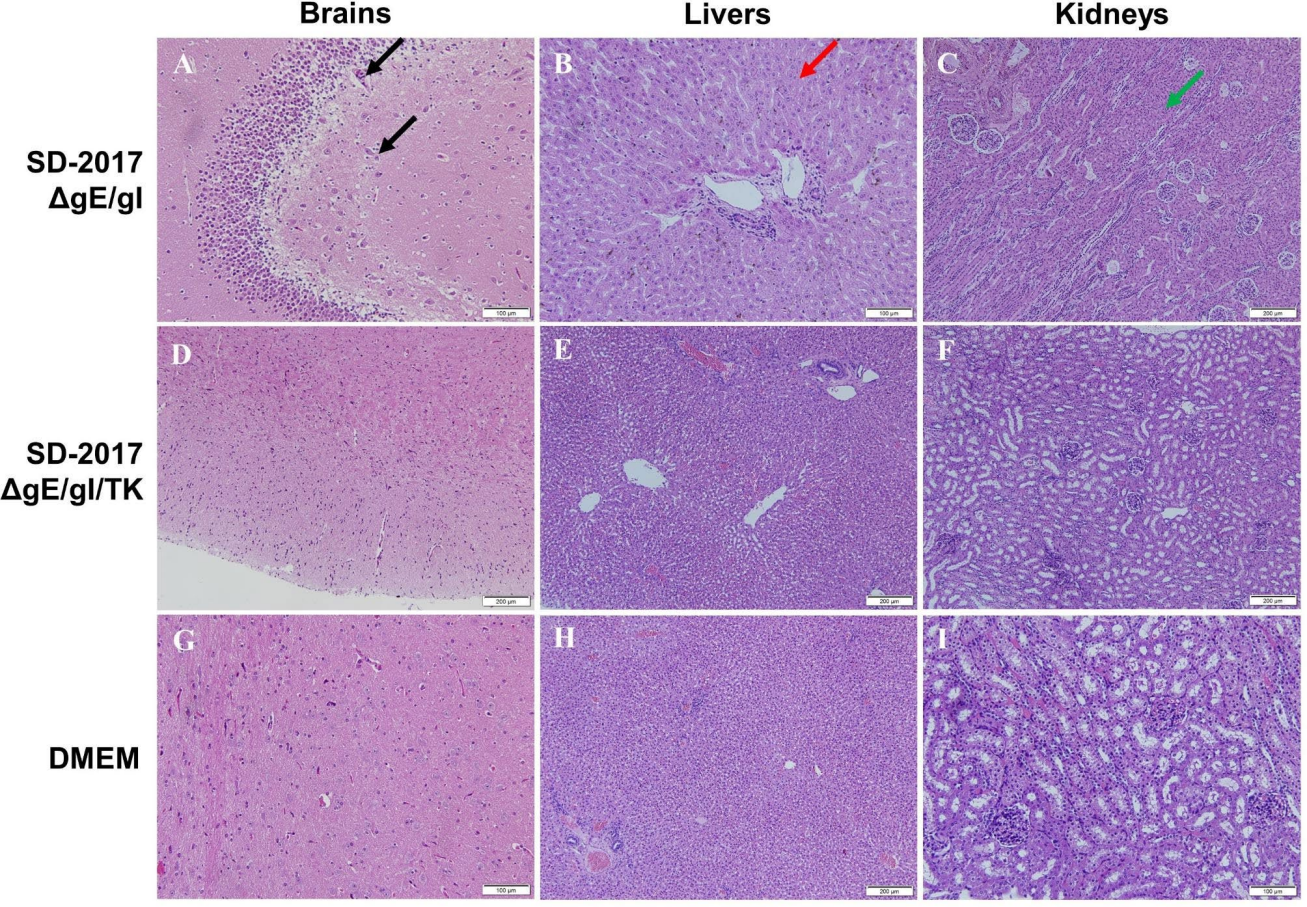
Pathogenicity of the genetically deleted PRV strains as indicated. Black arrow, widening of brain perivascular spaces; Red arrow, swollen and necrotic liver cells; Green arrow, swollen renal tubular epithelial cells with reduced lumens (atresia)

The live vaccine preparations were examined for lethality in rabbits and compared with the virulent SD-2017 strain (LD_50_ 3.16 × 10^2^ TCID_50_). The gE/gI gene deletion strain produced an LD_50_ of 7.94 × 10 TCID_50_ and the further deletion of the TK generated an LD_50_ of > 10^6^ TCID_50_. Rabbits that survived the observation period were euthanized and brains, livers and kidneys were examined for pathological changes. The SD-2017ΔgE/gI group displayed a mild widening in brain perivascular spaces, swollen and necrotic liver cells, and swollen renal tubular epithelial cells. In contrast, there were no obvious lesions in the SD-2017ΔgE/gI/TK and DMEM control groups (Fig. 4).

### Humoral immune response following immunization

**Fig. 5:**
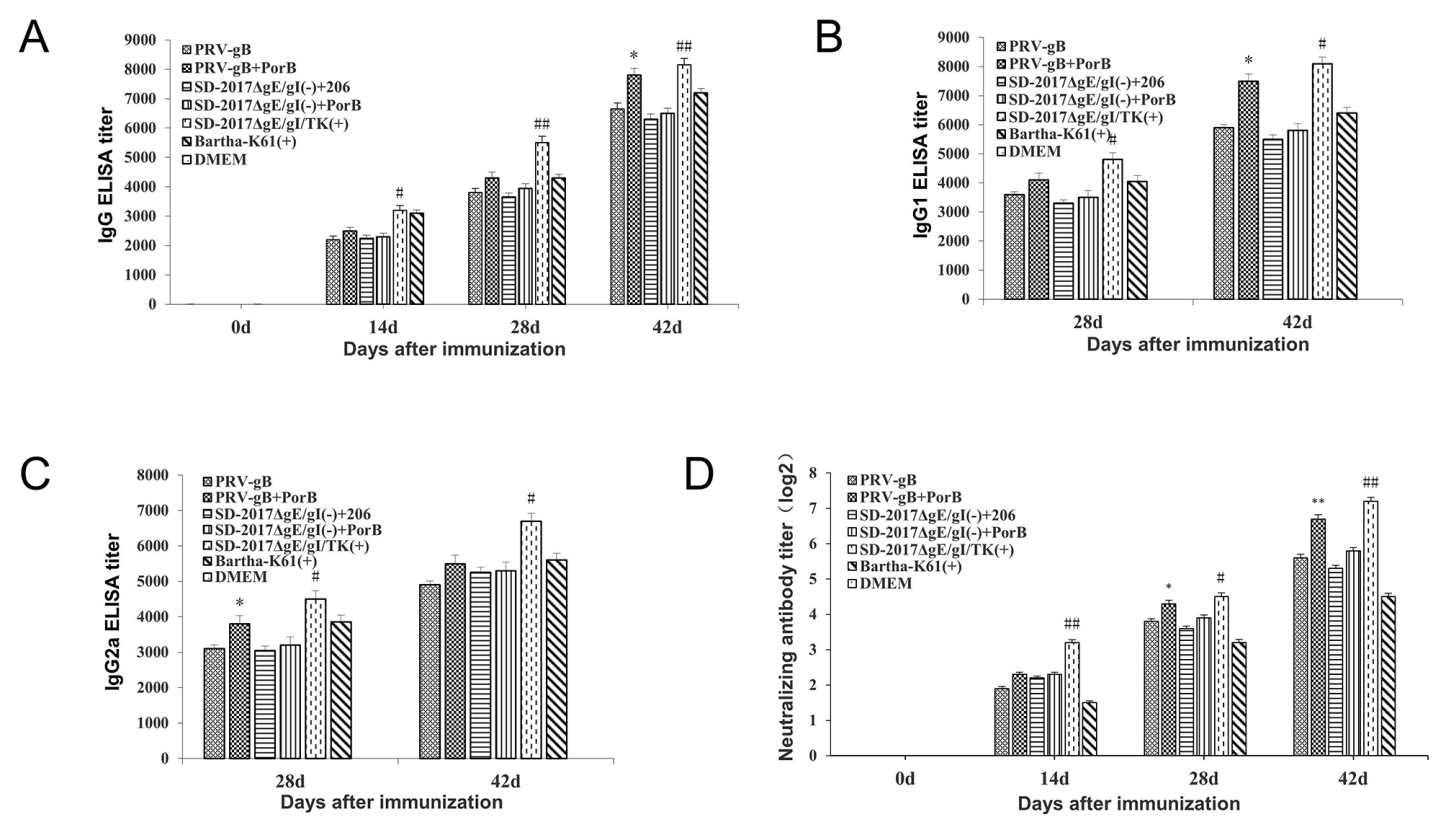
Antibody titers in immunized rabbits. **(A)** gB-specific IgG from serum samples at 14, 28 and 42 dpi. **(B)** PRV-specific IgG1 and **(C)** IgG2a titers at 28 and 42 dpi **(D)** Neutralizing antibody titers against PRV SD-2017 strain expressed as the log_2_ of the reciprocal of the highest serum dilution when PK15 cell infection was inhibited. n = 10. *, p < 0.05; **, p < 0.01; #, p < 0.05; ##, p < 0.01. (+), live vaccine; (-), inactivated vaccine. *, PRV-gB + PorB and SD-2017ΔgE/gI(-) + 206 groups; #, SD-2017ΔgE/gI/TK(+) and SD-2017ΔgE/gI(-) + 206 groups. 206, ISA 206 adjuvant

Humoral immunity was then evaluated by measuring total Ig and IgG1 and IgG2a isotypes in serum samples from animals vaccinated with the attenuated strains and the protein subunit vaccines (Fig. 5A). All groups with the exception of the DMEM controls produced PRV-specific IgG at 14, 28 and 42 dpi, although the live vaccine group produced higher levels at 14 dpi compared with the inactivated vaccine groups. In addition, by 42 dpi the IgG antibody titers of rabbits immunized with the PRV-gB + PorB subunit and SD-2017ΔgE/gI/TK(+) live attenuated vaccine were significantly higher than that of other groups (Fig. 5A). In particular, at 28 and 42 dpi IgG1 and IgG2a titers were elevated using PRV-gB + PorB, SD-2017ΔgE/gI/TK(+) as well as Bartha K61 (+) (Fig. 5B C). The results demonstrated that both the subunit and live attenuated vaccines effectively promoted humoral immune responses.

Neutralizing antibodies are an additional measure of vaccine effectiveness, so we further measured levels of PRV neutralizing antibodies for each vaccine group. All vaccine groups displayed SD-2017 strain-specific neutralizing antibody responses at 14, 28, and 42 dpi. The antibody titers of PRV-gB + PorB and SD-2017ΔgE/gI/TK(+) were significantly higher than the others at 28 and 42 dpi. In addition, compared with other groups, the attenuated live vaccine SD-2017ΔgE/gI/TK(+) significantly increased the titer of neutralizing antibodies at 14 dpi (Fig. 5D). These results demonstrated that the recombinant protein PRV-gB + PorB and the attenuated live vaccine SD-2017ΔgE/gI/TK(+)could effectively increase the titer of virus-neutralizing antibodies.

### Cellular immune response induced by PRV immunization

**Fig. 6:**
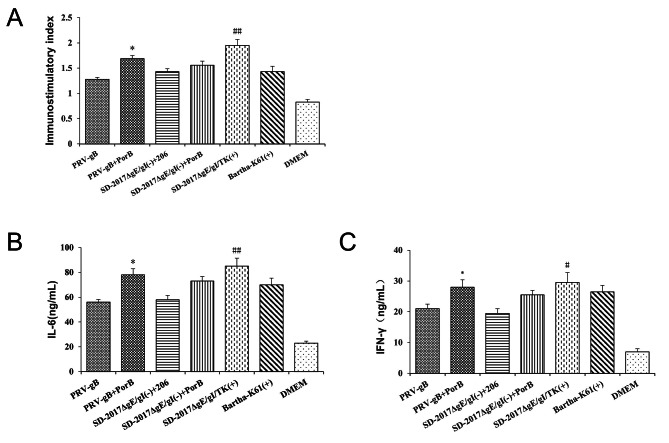
Cellular immune response analysis of immunized rabbits. **(A)** Lymphocyte proliferation test at 42 dpi **(B)** IFN-γ and **(C)** IL-6 levels in the supernatants of stimulated T cells at 42 dpi. n = 10. See Fig. 5 for abbreviations

In order to study the cellular immune response, the proliferation index (SI) of peripheral blood lymphocytes of rabbits were measured at 42 dpi. The differences in the stimulation index (SI) between the immunized groups and the DMEM controls were significant (p < 0.01) (Fig. 6A). In addition, the PRV-gB + PorB and SD-2017ΔgE/gI/TK(+) groups showed significantly higher SI values than the other groups. The IL-6 and IFN-γ cytokine levels, which are associated with Th2 bias and the Th1 cell response, respectively, were also increased in all vaccine groups (Fig. 6B C). Moreover, the IL-6 and IFN-γ levels for PRV-gB + PorB and SD-2017ΔgE/gI/(-) + PorB groups were significantly higher than the PRV-gB or SD-2017ΔgE/gI/(-) + 206 groups. These data demonstrated that the combined immunization of PorB protein significantly enhanced the cellular and humoral immune responses.

### Immune protection of vaccines against PRV variant strains

**Fig. 7:**
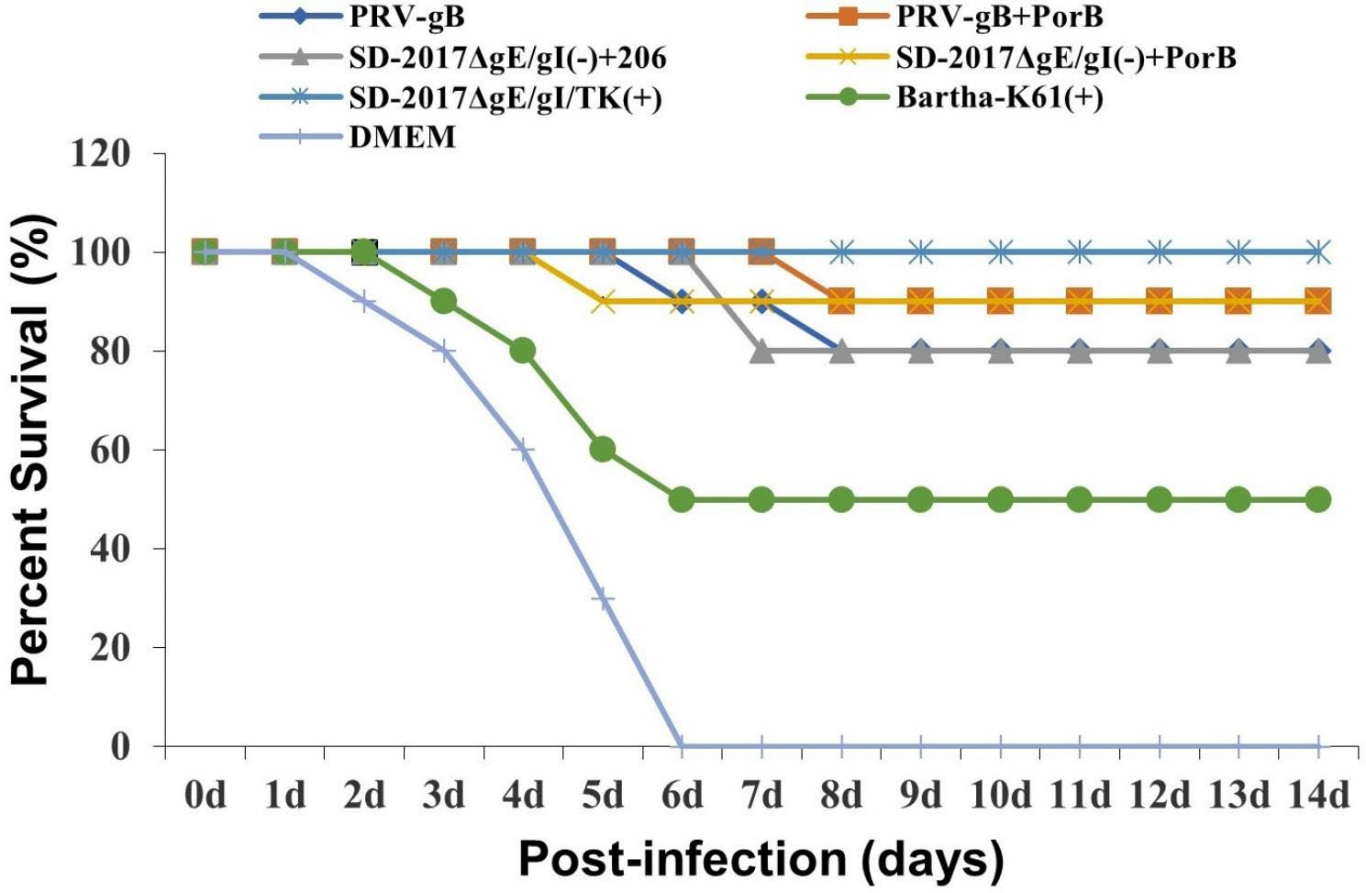
Protective immunity to rabbits intranasally infected with virulent PRV SD-2017 strain. Three weeks after the second immunization, rabbits in 7 groups were intranasally challenged with 100 LD_50_ of PRV SD-2017 strain. Percentage survival on the indicated days was calculated as: survival rate (%) = (numbers of mice surviving/numbers of total rabbits) × 100%. (+), live vaccine. (-), inactivated vaccine. 206, ISA 206 adjuvant

In order to evaluate the immune protective effect against the virulent PRV variant strains, rabbits were intranasally inoculated with 100 LD_50_ of the virulent SD-2017 strain. During the 14-day monitoring period, the rabbits immunized with the attenuated live vaccine SD-2017ΔgE/gI/TK(+) showed a 100% protection rate, while rabbits vaccinated with PRV-gB + PorB and SD-2017ΔgE/gI(-) + PorB showed 90% protection (Fig. 7). Rabbits immunized with recombinant protein PRV-gB and SD-2017ΔgE/gI(-) + 206 showed a protection rate of 80% (Fig. 7).

**Fig. 8:**
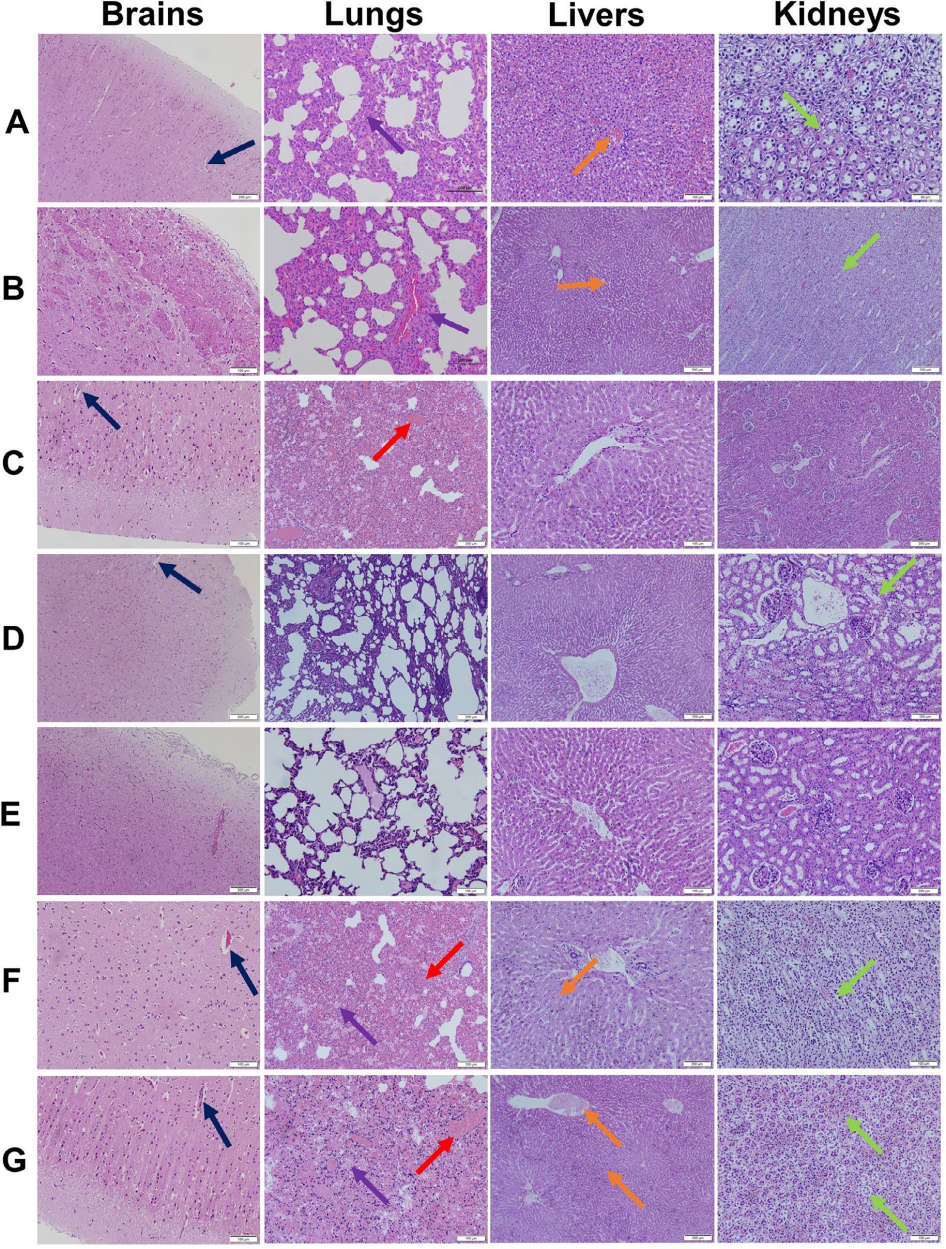
Histopathological observation of tissue slices form rabbits in the indicated vaccination groups at 14 d post-challenge. **(A)** PRV-gB, **(B)** PRV-gB + PorB, **(C)** SD-2017ΔgE/gI(-) + 206, **(D)** SD-2017ΔgE/gI(-) + PorB, **(E)** SD-2017ΔgE/gI/TK(+), **(F)** Bartha K61(+), **(G)** DMEM. HE, hematoxylin and eosin staining. 206, ISA 206 adjuvant. Scale bar, 100 or 200 μm. Black arrow, widening of brain perivascular spaces; Red arrow, lung congestion; Purple arrow, lung edema; Yellow arrow, liver congestion and swollen and necrotic cells; Green arrow, renal hemorrhage, congestion and swelling of some glomeruli

We additionally examined these test animals for particular pathologic signs (Fig. 8). The perivascular space in the brain animals immunized with DMEM and Bartha K61(+) was obviously enlarged and accompanied by neurophagy (Fig. 8 black arrow). There was also congestion and edema in the lungs, congestion in the liver including swelling and necrosis of some liver cells, hemorrhaging in the kidney, necrosis and shedding of some renal tubular epithelial cells. In contrast, we found no obvious histopathological lesions or pathological damage in rabbits immunized with SD-2017ΔgE/gI/TK(+), PRV-gB + PorB. These data demonstrated that the two novel vaccines constructed above provided efficient protection against PRV infection in rabbits.

## Discussion

Vaccination is one of the most effective methods to prevent and control PR [18]. At present, the most widely used vaccine strain Bartha-K61 has a 3,489 bp deletion at positions 122,560 − 126,049, which includes part of the gI gene, the full gE and US9 genes, and part of the US2 gene [22]. However, since 2011, despite vaccination measures, outbreaks of PR by new highly pathogenic PRV variants with unique molecular characteristics have occurred throughout China [6, 23, 24]. In the current study, we developed a gI/gE double gene deleted inactivated vaccine and a gI/gE/TK triple-gene deleted live vaccine based on the SD-2017 variant strain. We have adopted the strategy of knocking out all the gE/gI and TK (UL23) genes that reduced the risk of PRV returning to strong virulence through recombination, and the vaccine can be used for the implementation of the DIVA (Differentiating Infected from Vaccinated Animals) protocol. However, the SD-2017ΔgE/gI strain can only be used for the preparation of inactivated vaccines due to its virulence to rabbits (LD_50_ = 7.94 × 10^4^ TCID_50_) after the deletion of the gE/gI gene. Rabbit vaccination and protection test results indicated that SD-2017ΔgE/gI/TK(+) and SD-2017ΔgE/gI(-) + PorB vaccines had a protection rate of 90–100% against a lethal challenge with the SD-2017 variant, and the immune effect was remarkable. A limitation of the current study is that the newly developed vaccine candidates were tested in an antigenically homologous challenge setting (vaccine derived from the same virus as the challenge virus) whereas the Bartha-K61 vaccine was tested in a heterologous challenge setting (vaccine and challenge virus antigenically different), which may have contributed to the differences in protection observed for Bartha-K61 versus the newly developed vaccine candidates.

Currently, PR is controlled with the use of active attenuated vaccines even though the long-lasting immunity they provide can be compromised by insufficient attenuation and genetic instability [25]. The recombinant risk of live attenuated PRV vaccine needs to be taken seriously. Recombination, by exchanging genomic segments, may modify the virulence of alpha-herpesviruses [26]. Therefore, we explored the use of subunit vaccines expressing key PRV antigenic proteins as a safe alternative to the attenuated live vaccines, avoiding the biosafety risks of using of whole pathogens. There are currently no commercially available PRV subunit vaccines, our study was conducted to examine the protective effects of the PRV glycoproteins gB, theprimary antigens induce PRV humoral and cellular immunity [16]. Previous studies have evaluated induction of cellular immunity via gB, gC and gD recombinant heterologous vector vaccines in mice and pigs [27–29], and the high levels of cell-mediated immunity they generated significantly decreased viral shedding [30]. In the current study, we focused on the gB subunit derived from the highly virulent SD-2017 variant strain combined with the dendritic cell targeting peptide (DCpep) as an intramolecular adjuvant and *N. meningitidis* PorB as a novel protein adjuvant. The PRV-gB + PorB subunit vaccine was constructed by expressing PRV gB-DCpep and PorB protein containing gp67 protein secretion signal peptide using the baculovirus system, and this particular strategy has not been previously reported.

Most genetically-engineered subunit vaccines are composed of two primary components: antigen and adjuvant. Dendritic cells (DC) are potent antigen presenting cells and natural adjuvant for induction of humoral and cellular immunity [31]. Therefore, we utilized of the DCpep (FYPSYHSTPQRP) dendritic cell targeting molecule to enhance antigen presentation [32, 33]. We additionally included the outer membrane protein (OMP) PorB of *N. meningitidis*, a naturally occurring Toll-like receptor 2 (TLR2) ligand that activates innate and adaptive immune cells while regulating cellular immune responses [34, 35]. Vaccines formulated with PorB with ovalbumin induced multiple antigen-specific antibody subclasses and effector molecules such as MIG, MCP-1, IP-10, MIP-1α, KC and IL-2. These lead to increased levels of Th1- and Th2-type cytokines following antigenic stimulation [36, 37]. The PorB protein can increase antigen accumulation by follicular dendritic cells (FDC) and this increases B cell affinity maturation [38]. In our study, we immunized rabbits with PorB and gB-DCpep fusion protein subunit vaccine, which resulted in elevated levels of PRV-specific IgG1 and IgG2a isotypes and significantly increased the titer of neutralizing antibody at 28 and 42 dpi, and increased Th1 and Th2 cytokine levels following antigen stimulation. The survival rate of immunized rabbits against SD-2017 strain lethal challenge was 90%, and these animals did not display any overt or obvious histopathological damage. Although the overall protection provided by the gB-PorB protein subunit vaccine was lower than that of the triple gene deletion live vaccine SD-2017ΔgE/gI/TK, it possessed a good safety profile compared with the PRV live vaccine. Because subunit vaccines have no risk of viral recombination. Our data indicated that the PRV gB-PorB subunit vaccine has important developmental value.

Interestingly, we also found that the combined immunization of rabbits with PRV antigen and PorB was superior to the combined immunization with the 206 adjuvant. IL-6 and IFN-γ levels of rabbits vaccinated with SD-2017ΔgE/gI/(-) + PorB vaccine were significantly greater than for SD-2017ΔgE/gI/(-) + 206 vaccine, as well as the protection against an SD-2017 strain challenge. These data indicated that co-immunization with PorB was consistent with a good immune stimulating effect.

## Conclusions

We evaluated the immunogenicity and clinical protective effects of novel PRV vaccines in rabbits. The PRV SD-2017ΔgE/gI/TK live attenuated vaccine and PRV-gB + PorB subunit vaccine provided protection against challenge with the PRV variant strain, and SD-2017ΔgE/gI/TK provided the best protection. Interestingly, we demonstrated that the recombinant PRV subunit vaccine was effectively generated by the DCpep fused with PRV gB antigen, and the *N. meningitidis* PorB protein as adjuvant enhanced protective immunity against PRV. The results suggest that PRV-gB + PorB vaccine may be a safe and effective subunit vaccine candidate for PRV. This study serves as an important reference for the development of gene-deleted and subunit vaccines against the PRV variants.

## Data Availability

Data are available on reasonable request.

## Abbreviations

PR: pseudorabies
PRV: pseudorabies virus
SuHV1: Suid Herpesvirus-1
DC: Dendritic cells
DCpep: DC-targeting peptide
Nm: *Neisseria meningitidis*
PorB: outer membrane pore proteins of *N. meningitidis*
EGFP: enhanced green fluorescent protein
MOI: multiplicity of infection
LD_50_: median lethal dose
MTS: cell proliferation assay
TCID_50_: median tissue culture infective dose
SPF: specific pathogen free
OMP: outer membrane protein
TLR2: Toll-like receptor 2
FDC: follicular dendritic cells
IL-6: interleukin- 6
IFN-γ: interferon-gamma
IgG: immunoglobulin G.
